# Cardiopulmonary Profile in Streptozotocin-Induced Type 1 Diabetic Rats during Systemic Endotoxemia

**DOI:** 10.1155/2013/494179

**Published:** 2013-02-25

**Authors:** Ching-Hsia Hung, Che-Ning Chang, Yu-Wen Chen, Yu-Chung Chen, Jann-Inn Tzeng, Jhi-Joung Wang

**Affiliations:** ^1^Department of Physical Therapy, National Cheng Kung University, Tainan 701, Taiwan; ^2^Department of Physical Therapy, China Medical University, Taichung 404, Taiwan; ^3^Division of Physical Therapy, Department of Physical Medicine and Rehabilitation, Cheng Hsin Rehabilitation Medical Center, Taipei 112, Taiwan; ^4^Department of Food Sciences and Technology, Chia Nan University of Pharmacy and Sciences, Tainan 717, Taiwan; ^5^Department of Anesthesiology, Chi-Mei Medical Center, Tainan 710, Taiwan; ^6^Department of Medical Research, Chi-Mei Medical Centre, Tainan 710, Taiwan

## Abstract

This study was designed to determine the severity of cardiopulmonary dysfunction during systemic endotoxemia in type 1 diabetes. Thirty-two adult male Wistar rats were randomly assigned to a control group or to a group treated with streptozotocin (STZ) to create an animal model of type 1 diabetes. Survival time and cardiovascular parameters were continually monitored in urethane anaesthetized animals receiving intravenous infusion of endotoxin (lipopolysaccharide (LPS)) or saline. We also determined arterial blood gases, lung injury, and tumor necrosis factor-alpha (TNF-**α**) levels in serum and bronchoalveolar lavage fluid. Before LPS administration, the mean arterial pressure in STZ rats was significantly higher than that in normal rats. After LPS injection, the heart rate drop significantly in STZ rats than that in the control group. Also, the increased levels of TNF-**α** in serum and lavage fluid after LPS treatment were significantly higher in STZ rats than those in normal rats. Survival time in STZ rats was shorter than that in normal rats after LPS application. Albumin content, wet/dry weight ratio of lung, and lung injury were indistinguishable between STZ and normal rats. These results indicate that the cardiopulmonary change which occurs during LPS-induced endotoxemia is minor in STZ-induced diabetic rats.

## 1. Introduction

Due to the lower level and impaired binding activity of cell-surface receptors on monocytes [1], a poorly controlled diabetic state increases susceptibility to infections such as endotoxemia [2]. In the diabetic patients, infection is more serious and difficult to eradicate [3]. However, the severity of cardiopulmonary dysfunction in type 1 diabetes during systemic endotoxemia remains unclear.

As a systemic infection, endotoxemia is mainly caused by the endotoxin (lipopolysaccharides (LPS) from gram-negative bacteria [4]. Lipopolysaccharides binds to CD14 receptor with LPS-binding protein when entering the mammalian bloodstream. As a result, nuclear factor-*κ*B is activated to induce monocytes, macrophages, and endothelial cells to release cytokines such as tumor necrosis factor-alpha (TNF-*α*) [5] and causes tissue damage [6]. Also, arterial pressure is decreased due to the dilation of peripheral vessels in rats during endotoxemia [7]. Consequently, a fatal syndrome of irreversible cardiovascular collapse and septic shock may follow [8]. TNF-*α* plays an important role in this endotoxin-induced shock because serum TNF-*α* level increases during lethal endotoxemia [9, 10]. In addition, acute respiratory distress syndrome, which is caused by acute lung injury, is another complication of endotoxemia. A great number of monocytes and macrophages are present in alveoli and release TNF-*α*, which damages pulmonary vessels and increases lung vascular/epithelial permeability. Such an occurrence contributes to lung edema and poor lung compliance. Hence, septic patients may suffer from critical shock and acute respiratory distress syndrome as a group experience high mortality.

The mortality from cardiovascular disorders in type 1 diabetes is 4-to-37 times higher than that in the general population [11]. Severely uncontrolled diabetic state may initiate pathologic events leading to the capillary leak of acute respiratory distress syndrome as well [12]. Therefore, we hypothesized that the cardiopulmonary dysfunction induced by systemic endotoxemia will be more marked in type 1 diabetes. In the present study, we used type 1-like diabetic rats to investigate hemodynamic dysfunction and lung injury after intravenous LPS infusion. Also, the changes in TNF-*α* in serum and lung lavage fluid were determined.

## 2. Materials and Methods

### 2.1. Animals

Ninety-six adult male Wistar rats (320 ± 20 g) were purchased from the Animal Center of National Cheng Kung University Medical College (Tainan city, Taiwan). They were housed in groups of four at an ambient temperature of 24 ± 1°C and maintained under a normal light-dark cycle (14:10 h; lights on at 6:00 am). Pelleted rat chow and tap water were available ad libitum. All protocols were approved by the Institutional Animal Care and Use Committee of National Cheng Kung University, Tainan, Taiwan. All experimental procedures were conducted in compliance with the National Institutes of Health's “Guide for the Care and Use of Laboratory Animals.” Adequate anesthetic level was maintained to abolish the corneal reflex and pain reflexes induced by tail pinching throughout the course of all experiments (approximately 8 h each) after a single intraperitoneal dose (1.4 g/kg) of urethane.

### 2.2. Experimental Groups

Animals were randomly assigned to four groups (24 rats for each group): (a) normal rats receiving 0.9% normal saline administration (NS), (b) normal rats receiving LPS administration (NL), (c) diabetic rats receiving normal saline administration (SS), and (d) diabetic rats receiving LPS administration (SL). An animal model of type 1 diabetes, streptozotocin-(STZ-) treated rats, was prepared by intravenously injecting rats with STZ (Sigma, St. Louis, MO) (60 mg/kg) via the femoral vein to irreversibly destroy pancreatic *β* cells after the animals had fasted for 72 h [13]. Rats with hyperglycemia (blood sugar >300 mg/dL) and polyuria were considered diabetic. At the 5th week after STZ administration, survival time, and cardiovascular parameters were continuously monitored in animals receiving intravenous infusion of LPS or saline. Fresh solutions of LPS (from *Escherichia coli* 0111:B4, Sigma Chemical Co.) were prepared in phosphate buffered saline (pH 7.40) at a concentration of 10 mg/mL for infusion (15 mg/kg) into the femoral vein to induce endotoxemia. 

Different subgroups of animals (8 rats for each subgroup) were used for each of the four experiments: (I) determination of survival rate in normal and diabetic rats after receiving an injection of LPS or saline; (II) determination of the change of hemodynamic parameters during endotoxemia and lung edema at 180 min after LPS or saline injection; (III) determination of arterial blood gas, lung protein leakage, TNF-*α* level in serum and bronchoalveolar lavage, and lung histopathology at 180 min after LPS or saline injection.

### 2.3. Measurement of Hemodynamic Parameters

Animals were anesthetized with an intraperitoneal injection of urethane (1.4 g/kg) and the right femoral artery was cannulated using polyethylene catheters (PE-50). After cannulation, animals were stabilized for 1 h without data collection. Mean arterial pressure (MAP) and heart rate (HR) were recorded using a polygraph (MP35, BIOPAC Systems Inc., Goleta, CA, USA) every 20 minutes from the arterial tube in the femoral artery throughout endotoxemia. 

### 2.4. Analysis of Arterial Blood Gas

In order to determine arterial pH, arterial partial pressure of O_2_ (PaO_2_), CO_2_ (PaCO_2_), and O_2_ saturation (SO_2_) of rats, 0.4 mL of arterial blood was sampled from the femoral artery 3 h after administration of LPS using a syringe rinsed with heparin. Samples were analyzed by blood gas analyzer (Synthesis1725, Diamond Diagnostics Inc., Hollinton, MA, USA). 

### 2.5. Determination of TNF-*α* in Serum and in Bronchoalveolar Lavage Fluid

For determination of TNF-*α* levels, venous blood samples were taken from rats at 3 h after administration of LPS or saline. Blood samples were allowed to clot for 30 minutes at room temperature and then were centrifuged for 20 minutes (2000 g, 4°C). The supernatants were harvested and stored at −70°C until measurements were taken. At the same time, bronchoalveolar lavage samples were collected by perfusing saline from the endotracheal tube. The concentrations of TNF-*α* were determined using a double-antibody sandwich ELISA (R&D Systems, Minneapolis, MN, USA) according to the manufacturer's instructions. The optical density of each well was determined by microplate photometers (Multiskan EX, 110–120 V, Thermo Fisher Scientific Inc., Waltham, MA).

### 2.6. Detection of Lung Vascular/Epithelial Permeability

Albumin content of the bronchoalveolar lavage fluid was determined to assess the damage to endothelial cells of the lung capillaries. The lavage samples were analyzed using a Bio-Rad protein assay system (Bio-Rad Hercules, CA) with bovine serum albumin (BSA) as the standard. The albumin content in the bronchoalveolar lavage fluid was calculated by dividing the albumin by dried weight of lung to evaluate pulmonary capillary/endothelial cell permeability.

### 2.7. Measurement of Lung Edema

Three hours after administration of LPS (or saline), the entire lungs were dissected, weighed (wet weight), and dried (at 63°C for 48 hours). Dry weight was also recorded. The wet/dry weight ratio of lung tissue was calculated to evaluate lung edema. 

### 2.8. Histopathologic Change of Lung

Rats were perfused with saline followed by 4% paraformaldehyde buffer at 180 min after LPS administration. Serial sections (4 *μ*m) of right upper lobe were stained with hematoxylin and eosin for microscopic evaluation. The characteristics of lung damage include vascular congestion, hemorrhage, polymorphonuclear leukocytes (PMN) infiltration, and edematous changes of alveolar wall [14]. Each characteristic was scored (0: normal; 1: mild; 2: moderate; 3: severe) by a pathologist and overall lung injury was further calculated according to the sum of the score. Moreover, the degree of leukocyte infiltration, which was assessed as the PMN/alveoli ratio, can stand for lung damage. We selected 10 randomly areas at high-power field (HPF, 400x) of each blind sample. Then alveoli and PMNs in each area were counted and PMN/alveolus ratio was expressed by dividing the sum of PMNs in 10 HPF fields by that of alveoli.

### 2.9. Statistical Analysis

Data are expressed as mean ± S.E.M. for each point. Statistical analysis was conducted using analysis of variance (ANOVA) for factorial experiments, and repeated-measures ANOVA (followed by Scheffe after hoc test) for comparing hemodynamic parameters. The Wilcoxon rank-sum test was used for analysis of survival time the Kaplan-Meier test for survival rates. Significant differences between groups were assumed to be present at values of *P* < 0.05.

## 3. Results

Blood glucose in diabetic rats was substantially (approximately 5-fold) and significantly (*P* < 0.001) higher than that in normal rats (361 ± 9 mg/dL versus 73 ± 3 mg/dL). Body weights in STZ-treated rats were significantly lower than those in controls (262 ± 10 gm versus 385 ± 9 gm, *P* < 0.05) at the 5th week after injection of STZ. 

### 3.1. Survival Time and TNF-*α* Levels

**Figure 1:**
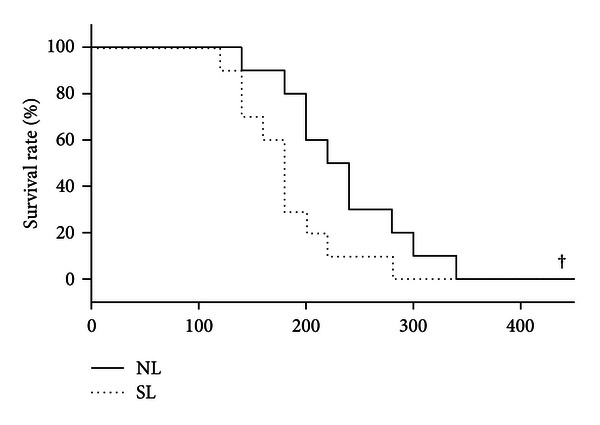
Effect of LPS administration (15 mg/kg, i.v.) on the survival rate of normal and diabetic rats. NL: normal rats receiving LPS administration; SL: STZ-treated rats receiving LPS administration. Bars represent mean ± S.E.M of 8 rats per group. ^†^ *P* < 0.05, compared with the NL group.

**Figure 2:**
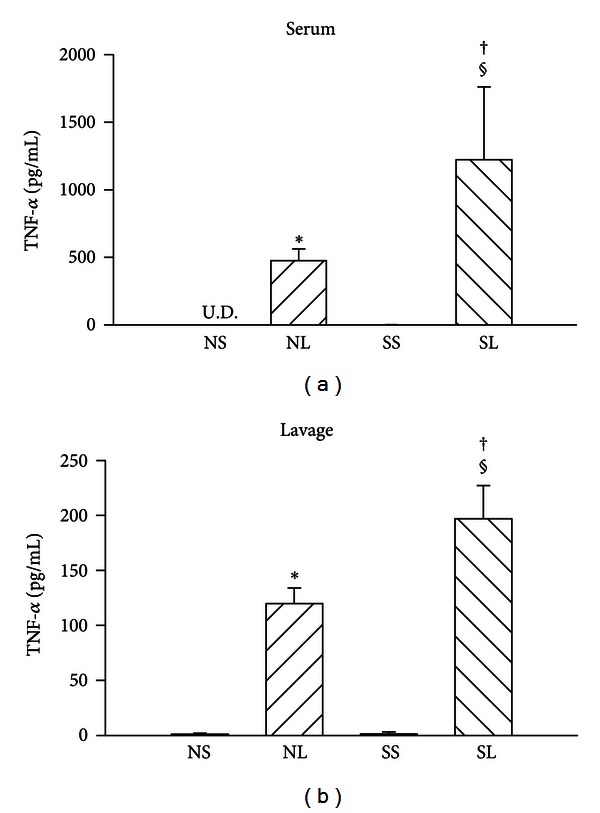
Serum and lavage fluid TNF-*α* levels in different groups of rats. NS: normal rats injected with saline; NL: normal rats injected with LPS; SS: STZ-treated rats injected with saline; SL: STZ-treated rats injected with LPS; U.D.: undetectable. Data are expressed as mean ± S.E.M (*n* = 8). *P < 0.05, compared with the NS group; ^†^ *P* < 0.05, compared with the NL group; ^§^ *P* < 0.05, compared with the SS group (one-way ANOVA).

After administration of LPS, the survival time of STZ-treated rats was markedly shorter than that of control rats (180.0 ± 14.6 min versus 224.4 ± 17.9 min, *P* = 0.027). Also, the survival rate of diabetic rats was significantly lower than the rate for control rats (Figure 1).  Figure 2 shows that TNF-*α* levels in serum and bronchoalveolar lavage fluid were significantly raised by administration of LPS in both normal and diabetic rats. Actually, the TNF-*α* levels for normal or diabetic rats were undetectable with saline treatment. Moreover, the LPS-induced increases of TNF-*α* levels in serum and bronchoalveolar lavage fluid were statistically higher in diabetic rats as compared with control normal rats.

### 3.2. Hemodynamic Alterations

**Figure 3:**
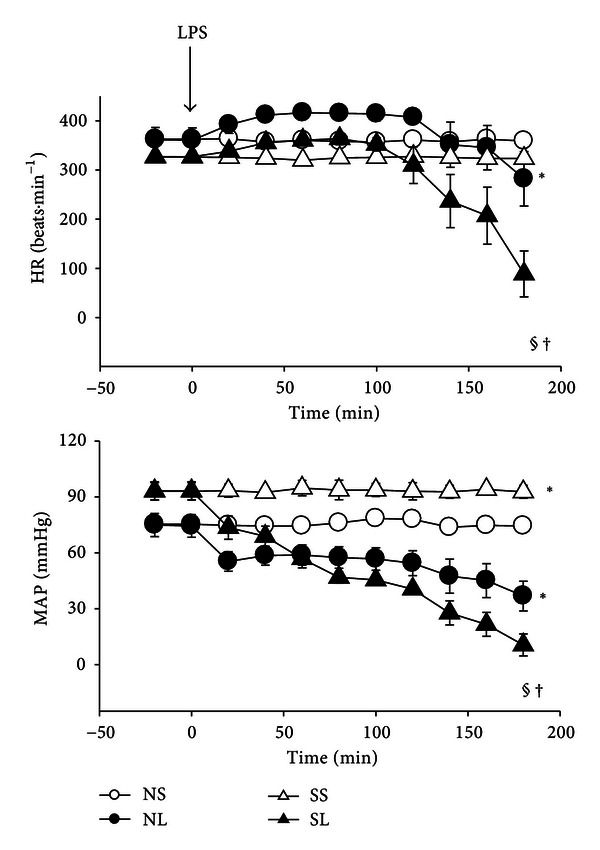
Changes in cardiovascular parameters in normal and diabetic rats during the administration of saline or LPS (15 mg/kg, i.v.). HR: heart rate; MAP: mean arterial pressure. Data are expressed as mean ± S.E.M (*n* = 8). *P < 0.05, compared with the NS group; ^§^ *P* < 0.05, compared with the SS group; ^†^ *P* < 0.05, compared with the NL group (repeated measures ANOVA).

The time course for changes in cardiovascular parameters in normal and STZ-treated rats after the administration of saline or endotoxin is shown in Figure 3. Mean arterial pressure was higher in diabetic rats than that in normal rats with saline administration. After LPS challenge, heart rate increased slightly and then dropped dramatically. Moreover, heart rate and mean arterial pressure were significantly decreased in diabetic rats than those in control ones.

### 3.3. Lung Injury

**Figure 4:**
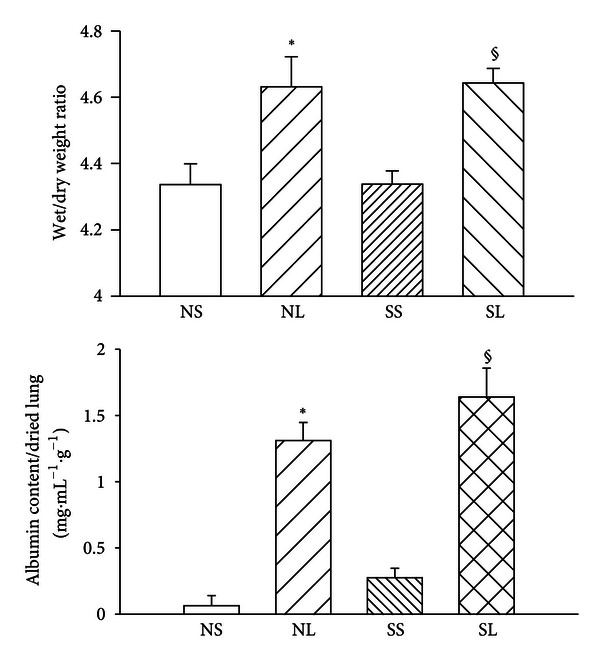
The albumin content in lung and lung wet/dry weight ratios in different groups after LPS administration. Albumin content in the bronchoalveolar lavage fluid was calculated as [(mg of albumin)/(mL of lavage fluid)]/(g of dried lung weight) after LPS administration (15 mg/kg, i.v.) in different groups. NS: normal rats injected with saline; NL: normal rats injected with LPS; SS: STZ-treated rats injected with saline; SL: STZ-treated rats injected with LPS. Data are expressed as mean ± S.E.M of 8 rats per group. *P < 0.05, compared with the NS group; ^§^ *P* < 0.05, compared with the SS group (one-way ANOVA).

**Figure 5:**
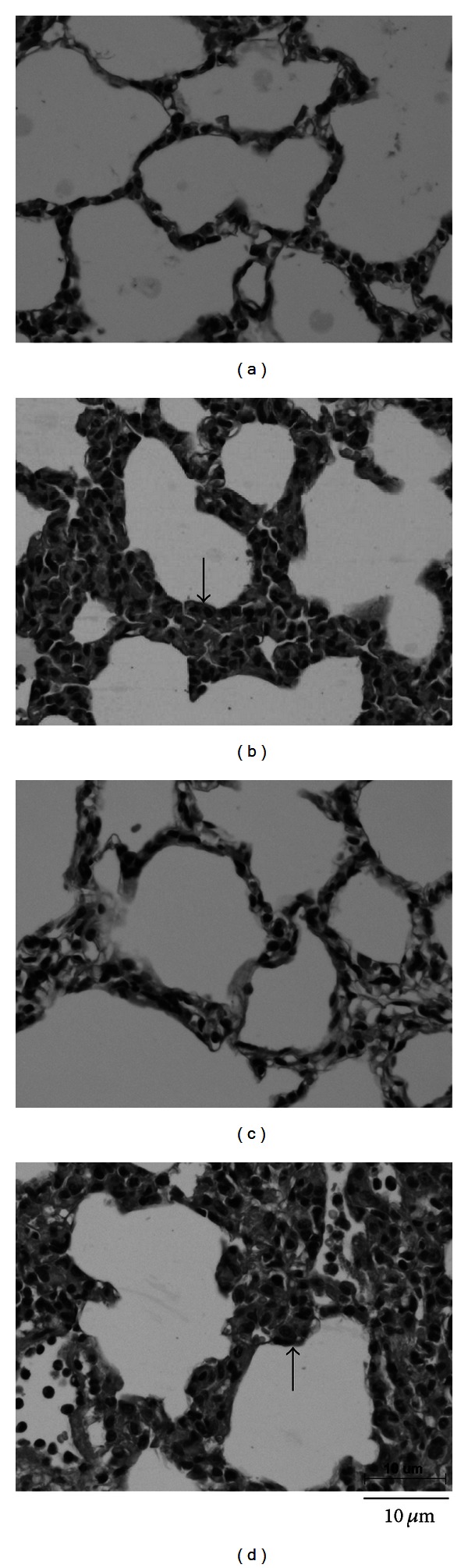
Hematoxylin and eosin-stained sections of right upper lobe from different groups of rats taken after 3 hours of LPS administration (HPF, 400x). (a) Normal rats injected with saline; (b) STZ-induced diabetic rats injected with saline; (c) normal rats injected with LPS; (d) STZ-induced diabetic rats injected with LPS. Arrows denote edematous changes of alveolar walls.

**Figure 6:**
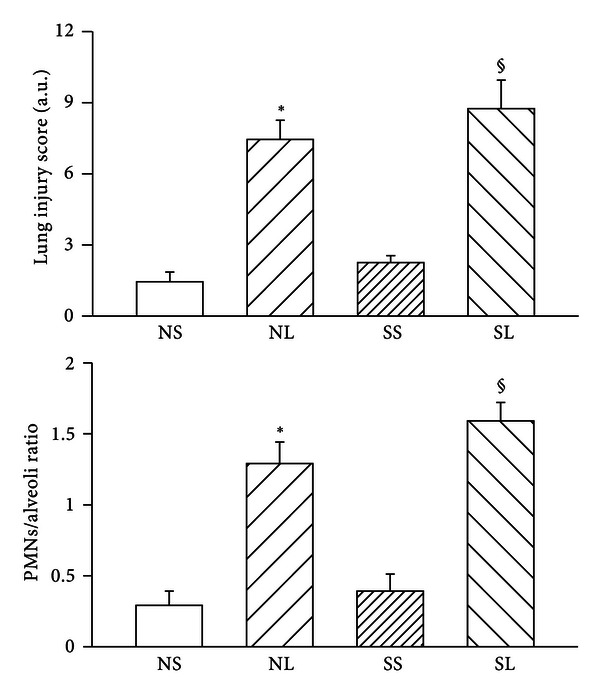
The lung injury score and PMNs/alveoli ratio in different groups after LPS administration. NS: normal rats injected with saline; NL: normal rats injected with LPS; SS: STZ-treated rats injected with saline; SL: STZ-treated rats injected with LPS. Data are expressed as mean ± S.E.M. *P < 0.05, compared with the NS group; ^§^ *P* < 0.05, compared with the SS group.

The ratio of wet lung weight to dry lung weight and the albumin content in lung increased significantly after LPS administration, and these parameters were indistinguishable between NL and SL groups (Figure 4). Histopathologic examination of the lungs demonstrated several changes after 3 hours of LPS administration (Figure 5). The interstitial spaces of aveoli became thickness with polymorphonuclear leukocytes infiltration as well as edematous changes of alveolar walls were showed in both normal and diabetic groups. Quantitative analyses of histology were shown in Figure 6. The lung injury score and PMNs/alveoli ratio were significantly increased in NL and SL groups compared with that of NS and SS groups. However, the values of lung injury score and PMNs/alveoli ratio are indistinguishable between SL and NL group. Baseline values for PaO_2_ and SaO_2_ for diabetic rats were significantly lower than those for control rats (Table 1). During endotoxemia, PaCO_2_ diminished significantly, whereas PaO_2_ and SaO_2_ were significantly greater in control and diabetic rats.

## 4. Discussion

In the present study, we demonstrated alterations in cardiopulmonary function in both control and type 1 diabetic rats during systemic endotoxemia. We found higher TNF-*α* levels in serum and bronchoalveolar lavage fluid in diabetic rats during systemic endotoxemia. Shorter survival times and the hemodynamic abnormalities observed in diabetic rats were worse after LPS challenge. However, the difference is minor from normal rats.

A previous report showed a lower survival rate with a higher TNF-*α* level during endotoxemia [15]. Multiple organ injury and irreversible cardiovascular collapse are induced in animals receiving recombinant human TNF [16]. Conversely, monoclonal antibodies against TNF-*α* and agents which antagonize LPS-induced production of TNF-*α* are effective in preventing LPS-induced lethality in vivo and in vitro [9, 17, 18]. Thus, TNF-*α* level seems to be related to survival rate. In the present study, the finding of a high level of TNF-*α* in the serum and bronchoalveolar lavage fluid of diabetic rats appears to be responsible for the higher mortality rate associated with LPS treatment.

Regarding hemodynamic parameters, our data show that the value of mean arterial pressure is higher in diabetic rats than control rats during saline administration. These changes are consistent with previous studies that used the STZ-induced diabetic model for chronic and continuous hemodynamic measurement [19, 20]. Those studies showed that the onset of hyperglycemia in the earliest stage of diabetes increases mean arterial pressure. Moreover, studies in patients with early uncomplicated type 1 diabetes also show an increase in arterial pressure during poor glycemic control [21, 22]. This increase is correlated with angiotensin II, due possibly to overstimulation of the renin-angiotensin system in response to hyperglycemia. In addition, endothelial dysfunction, which can be seen in type 1 diabetic patients, was found in the rat's aorta at the 4th week after STZ injection [23]. 

Heart rate was reported to be increased in order to compensate for the endotoxin-induced low blood pressure [24], which is consistent with our results during the early stages of endotoxemia. However, later on, the animals were unable to compensate, and heart rate dropped dramatically. Our data demonstrated that blood pressure drops in diabetic rats significantly than that in normal rats after LPS challenge whereas the difference among LPS-treated normal and diabetic groups was minor. The present results are in good agreement with those of previous findings showing that endotoxin administration to diabetic animals resulted in more severe cardiovascular dysfunction than in nondiabetic animals [25]. 

High levels of TNF-*α* were found in serum and lung in rats with endotoxemia, and more albumin was present in endotoxemic lungs than in control ones [26]. Yoshinari et al. found numerous polymorphonuclear cells in thickened alveolar interstitial tissue during endotoxemia [27]. The TNF-*α* released by polymorphonuclear cells damages the pulmonary vessels and increases alveolar-vessel permeability which causes lung protein leakage. Therefore, the lung injury is associated with elevated cytokines. Some evidence has shown that albumin content in the bronchoalveolar lavage fluid increases significantly, and lung compliance is reduced after endotoxin treatment [28]. In this study, we showed that diabetic rats have significantly higher TNF-*α* levels in lavage fluid from the lungs, but not greater albumin content and lung edema during endotoxemia. Rojas et al. have shown that TNF-*α* and lung edema have different time courses during endotoxemia [29]. After 6 hours of LPS administration, increased TNF-*α* brings about lung injury. The TNF-*α* content was significantly higher at 24 h than at 6 h after LPS injection. However, the degree of lung edema was lower at 24 h than at 6 h. There seemed to be a TNF-*α* threshold for causing lung injury and another, much higher, threshold for inducing more serious damage. In contrast, previous studies showed that diabetes may be associated with a lower-than-average risk of acute lung injury after intratracheal instillation of LPS [30], and the concentrations of TNF-*α* in the bronchoalveolar lavage fluid of intratracheal LPS-treated diabetic rats were markedly reduced [2]. It is possible that intratracheal instillation of LPS and intravenous LPS administration cause different pulmonary responses in diabetic rats. In addition, the response to LPS may be different between STZ-induced and alloxan-induced diabetic rats.

The present results show that PaCO_2_ decreases and PaO_2_ increases significantly during endotoxemia. A previous study showed that endotoxemia causes large changes in the respiratory system [31]. Endotoxin upregulates reactive oxygen species, TNF-*α*, and many other mediators. These are harmful mediators and are associated with respiratory muscular dysfunction [32]. Increases in vasodilators and vasoconstrictors that are induced by endotoxemia cause vessel dysfunction and redistribute blood flow. The insufficient blood flow for several organs slows down metabolic rate and decreases oxygen consumption. As a result, the generation of CO_2_ decreases [33, 34].

There are some limitations to our study. The higher mortality rate of STZ-treated rats may correlate with higher levels of TNF-*α* after LPS administration, but the mechanisms underlying the differences in survival rate between diabetic rats and nondiabetic rats are still unclear. To explore whether TNF-*α* plays a critical role during endotoxemia in diabetic rats, it will be necessary to study in vivo blockade of TNF-*α*. Moreover, it is important to determine whether glycemic normalization in diabetic rats can reduce TNF-*α* levels and improve survival after LPS injection. Also, further studies will be necessary to determine the responses of other inflammatory mediators (e.g., nitric oxide, Macrophage inflammatory protein-1*α*, etc.) during endotoxemia.

In conclusion, the difference of cardiopulmonary dysfunction between normal and STZ-induced diabetic rats was minor whereas the TNF-*α* levels in serum and bronchoalveolar lavage fluid were increased more markedly in diabetic rats receiving an LPS challenge. 

## Figures and Tables

**Table 1:** Effects of LPS administration (15 mg/kg, i.v.) on arterial blood gas in normal and diabetic rats.

	pH	PaCO_2_ (mmHg)	PaO_2_ (mmHg)	SaO_2_ (%)
NS	7.38 ± 0.01	42.03 ± 1.08	106.95 ± 2.28	97.82 ± 0.17
NL	7.40 ± 0.02	30.66 ± 1.99^a^	122.09 ± 5.93^a^	98.77 ± 0.21^a^
SS	7.41 ± 0.01	41.96 ± 0.92	91.40 ± 2.64^a^	96.93 ± 0.37^a^
SL	7.43 ± 0.01	30.63 ± 1.58^c^	106.50 ± 3.55^b,c^	97.96 ± 0.23^b,c^

PaCO_2_: arterial carbon dioxide tension; PaO_2_: arterial oxygen pressure tension; SaO_2_: oxygen saturation. Data are expressed as means ± S.E.M of 8 rats per group. ^a^ *P* < 0.05, compared with the NS group; ^b^ *P* < 0.05, compared with the NL group; ^c^ *P* < 0.05, compared with the SS group (one-way ANOVA).

## Abbreviations

HPF: High-power field
HR: Heart rate
LPS: Lipopolysaccharide
MAP: Mean arterial pressure
NL: Normal rats injected with LPS
NS: Normal rats injected with saline
SL: STZ-induced diabetic rats injected with LPS
SS: STZ-induced diabetic rats injected with saline
STZ: Streptozotocin
TNF-*α*: Tumor necrosis factor-alpha.
